# Drying Kinetics and Quality of Whole, Halved, and Pulverized Tiger Nut Tubers (*Cyperus esculentus*)

**DOI:** 10.1155/2021/8870001

**Published:** 2021-04-03

**Authors:** Ernest Ekow Abano, Joshua Akanson, Nazir Kizzie-Hayford

**Affiliations:** ^1^Department of Agricultural Engineering, School of Agriculture, College of Agriculture and Natural Sciences, University of Cape Coast, Cape Coast, Ghana; ^2^Department of Biochemistry, School of Biological Science, College of Agriculture and Natural Sciences, University of Cape Coast, Cape Coast, Ghana

## Abstract

The objective of this study was to provide the optimum drying conditions to produce high-quality dried tiger nuts using hot-air drying. For this, we evaluated the effect of the whole, halved, and pulverized tiger nuts and air temperature (50 to 70°C) on the drying kinetics and quality of tiger nuts. The drying process generally followed a constant rate in the first 3 hours and a falling regime. We found the optimum drying conditions for tiger nuts to be crushed before convective hot-air drying at a temperature of 70°C. At this optimum condition, the predicted drying time, vitamin C content, reducing sugars, browning, brightness, redness, and yellowness was 780 min, 22.9 mg/100 mg dry weight, 157.01 mg/100 g dry weight, 0.21 Abs unit, 56.97, 1.6, and 17.0, respectively. The tiger nut's reducing sugars increased from the 130.8 mg/100 dry weight in the raw tiger nuts to between 133.11 and 158.18 mg/100 dry weight after drying. The vitamin C degradation rate was highest in the uncut tiger nuts (32-35%) while in the halved and the pulverized samples, it was between 12 and 17%. The crushed samples' effective moisture removal increased between 5.6- and 6.75-fold at the different air temperatures than that of the intact tiger nuts. The activation energy was 18.17 kJ/mol for the unbroken, 14.78 kJ/mol for the halved, and 26.61 kJ/mol for the pulverized tiger nut samples. The model MR = 0.997 exp(−0.02*t*^1.266^) + 0.0000056*t* was the most suitable thin-layer drying model among the models examined for convective hot-air drying of tiger nuts. It is advisable to crush tiger nut before hot-air drying to produce better-quality flour for making milk beverages, cakes, biscuits, bread, porridge, and tiger nut-based breakfast cereals.

## 1. Introduction

Tiger nuts (*Cyperus esculentus*) are a tuber crop belonging to the family Cyperaceae, which is cultivated worldwide [1] The black, brown, and yellowish-brown tubers are the cultivars commonly produced in many parts of the world including Ghana. It is in the same family as the sedges and usually called earth almond, yellow nut sedge, and rush nut in many parts of the world [2]. In Ghana, it is widely known as “Atadwe” while in Spain, it is called “chufa.” The tubers contain an appreciable amount of dietary fibre, minerals such as potassium, phosphorus, calcium, magnesium, zinc, copper, and vitamins C and E, and essential fatty acids like myristic acid and oleic and linoleic acid [3]. Studies show that tiger nut extract and dietary fibre can effectively treat and prevent many diseases, including constipation, colon cancer, coronary heart disease, obesity, diabetes, and gastrointestinal disorders, and weight gain [1]. Many researchers value tiger nut for its aphrodisiac properties and copulatory functions [4, 5]. The tubers contain more than 60% of monounsaturated fatty acid, similar to that of olive oil [6]. The phytosterols that distinguish tiger nuts from olive are much richer in tiger nuts [7]. In Ghana, tiger nut is washed to remove dirt and spread on tarpaulins in the open sun to gradually lose their moisture and dry. Sun-died tiger nuts can last up to three months [8] accompanied by shrinkage, skin wrinkles, and hard nut texture. In Ghana, tiger nut tubers are typically consumed fresh or as tiger nut milk, which substantially affects its storage preservation and quality. Safe keeping and selling of fresh tiger nuts are confined to local markets because the tubers have short shelf life after harvest. Growth of mold, rot, and yeast and microbial contamination are typically associated with fresh tubers that are packed and stored under tropical conditions due the relatively high moisture content and sweetness of tiger nut tubers [7]. High postharvest losses thus characterize the tiger nut value chain if not consumed within a short time. Therefore, one of the appropriate ways to reduce postharvest losses and prevent rot and bacterial infections and secure quality and nutritional integrity is to process raw tiger nut into dried form or flour, which can be used to make milk beverages, cakes, biscuits, bread, porridge, and tiger nut-based breakfast cereals [9]. Among the novel technologies, the convective hot-air drying is the commonest in industrial dehydration to produce quality dried products [9]. Conventionally, drying the whole tiger nut is difficult, energy-intensive, and time-consuming and often produces relatively high moisture trapped within the tissues. These disadvantages are due to the tiger nuts tough skin texture that prevents rapid removal of moisture from the tissues to safe moisture levels within a short time to ensure long-term storage and fine flour production [2]. A modification of the tiger nut is thus required to enhance the drying rate and produce quality products. In Ghana, tiger nut consumers have strong preference for the brown variety because of its sweeter taste and softer skin toughness than the black type, making it an ideal variety for dehydrated snacks. Also, there is no known study on the drying kinetics and thin-layer drying modelling on tiger nuts to the best of the authors' knowledge. For commercial production of dried products, modelling plays an essential role in drying technology to select the most appropriate drying model and optimal working conditions for processing the product [10]. Therefore, the objective of this study was to provide the optimum drying conditions to produce high-quality dried tiger nuts using the convective hot-air drying. For this, the impact of air temperature on the drying kinetics and quality of whole, halved, and pulverized brown tiger nut tubers was investigated. The study in addition thus selected the most suitable drying model for hot-air drying of a total, split, and pulverized tiger nut.

## 2. Materials and Methods

### 2.1. Sample Preparation

The fresh tiger nut tuber for the drying experiment was obtained from Twifo Praso market in Twifo-Atti Morkwaa District of the central region. It was transported in polythene bags to the laboratory of the School of Agriculture, University of Cape Coast, A. G. Carson Technology Centre. The samples were visually selected based on uniform colour, size (10.41 ± 0.91 mm thick), and spherical geometry for drying. The initial moisture content of 47.43 kg H_2_O/kg d.m was found by weighing 5 g crushed samples and oven-dried at 105°C for 24 hrs, using the analytical method reported by AOAC [11]. We manually washed the tiger nuts and prepared them into three different forms: whole, halved by cutting into two with a stainless-steel knife along the longitudinal axis, and pulverized with a pulse kitchen blender (Ninja, 110 V, 1001 W, Needham, Massachusetts, USA) into coarse grits. The thickness of the whole and halved pieces was 10.41 ± 0.91 mm, while the crushed was 21.20 mm. The samples were kept in a refrigerator at 4°C to slow down the physiological and chemical changes. Before subjecting it to drying, the samples were allowed to warm up to room temperature conditions.

### 2.2. Drying Equipment and Drying Procedure

We subjected the whole, halved, and pulverized tiger nuts to convective hot-air drying using a cabinet food dehydrator (Klarstein, 220 V/50 Hz 1000 W, Berlin, Germany). Three temperature levels of 50, 60, and 70°C at an air circulation rate of 1.0 m/s for the hot-air drying experiment were used. Before the drying experiment, the dryer was run idle for 30 min to attain steady-state conditions for the desired hot-air temperature. The sample's mass was recorded every 30 min during the initial stages of drying and later changed to 1 hour until constant weight was observed. Weighing was done with a high-precision digital electronic balance (Great Wall Instruments, 5000 g, 0.01 g, 9 V, Jie Yang, Guangdong, China) to an accuracy of 0.01 g within 10 seconds.

### 2.3. Experimental Design

We used a 2-factor, three-level factorial design in a completely randomized design to plan the drying experiments using Minitab version 17. Air temperature and the form of the tiger nuts were chosen as the two design factors. We based the two factors on the state of the tiger nut and the hot-air temperature and their chosen levels on preexperimental trials. The analysis of variance was performed at a probability level of 95% to investigate the significance of the levels of the factors.

### 2.4. Drying Rate

The drying rate (DR) from the tiger nuts was computed using

(1) $$\begin{array}{c} \text{DR}=\frac{M_{t+∆t}-M_{t}}{∆t}, \end{array}$$

where DR is the drying rate (kg H_2_O/min) and *M*_*t*_ and *M*_*t*+dt_ are the moisture content in kg H_2_O/kg d.mF at drying time (*t*) and change in drying time ∆*t*.

### 2.5. Determination of Moisture Diffusivity and Activation Energy

We used Fick's modified second law of diffusion (equation (2)) to describe the moisture movement through food materials during drying to calculate the effective moisture removal, considering constant moisture diffusivity, infinite slab geometry, and uniform initial moisture distribution.

(2) $$\begin{array}{c} \text{MR}=\frac{8}{\pi^{2}}\overset{\infty }{\underset{n=0}{\sum }}\frac{1}{\left(2n+1\right)2}\;\exp\left[-\frac{{\left(2n+1\right)}^{2}\pi^{2}D_{\text{eff}}\;t}{4L^{2}}\right]. \end{array}$$

*D*_eff_ is the effective moisture diffusivity (m^2^/s) and *L* is half the thickness of the sample (m). Equation (2) simplifies to equation (3) for long drying times:

(3) $$\begin{array}{c} \text{MR}=\frac{8}{\pi^{2}}\;\exp\left[-\frac{{\left(2n+1\right)}^{2}\pi^{2}D_{\text{eff}}\;t}{4L^{2}}\right]. \end{array}$$

We obtained the *D*_eff_ for the tiger nut samples from the slope (*K*) of In(MR)'s graph against the drying time *t*. In(MR) versus *t* results in a straight line with a negative slope, and *K* is related to *D*_eff_ by

(4) $$\begin{array}{c} K=\frac{\pi^{2}D_{\text{eff}}\;}{4L^{2}}. \end{array}$$

*D*_eff_ is related to temperature by the Arrhenius equation (shown below):

(5) $$\begin{array}{c} D_{\text{eff}}=D_{0}\exp\left[-\frac{E_{\text{a}}}{R\left(T+273.15\right)}\right], \end{array}$$

where *D*_0_ is the constant in the Arrhenius equation (m^2^/s), *E*_a_ is the activation energy (J/mol), *T* is the temperature of hot air (°C), and *R* is the universal gas constant (8.31451 Jmol^−1^ K^−1^). The Arrhenius equation can be rearranged into the following form:

(6) $$\begin{array}{c} \text{In}\left(D_{\text{eff}}\right)=\text{In}\left(D_{0}\right)-\frac{E_{\text{a}}}{R\left(T+273.15\right)}. \end{array}$$

We computed the activation energy for moisture removal from the slope of the graph of In(*D*_eff_) against 1/(*T* + 273.15).

### 2.6. Mathematical Modelling of Drying Data

We expressed the drying kinetics of tiger nut slices in terms of empirical models. The modelling was based on the assumptions that the resistance to water movement to the surface of the sample is negligible and that the resistance to water evaporation is concentrated on the surface of the sample. This phenomenon is similar to Newton's law of cooling. The experimental data obtained were plotted in the form of a dimensionless moisture ratio (MR) against drying time in minutes. The MR of the tiger nut slices was determined using

(7) $$\begin{array}{c} \text{MR}=\frac{M-M_{\text{e}}}{M_{\text{o}}-M_{\text{e}}}. \end{array}$$

MR is the moisture ratio, *M*_i_ is the initial moisture content, *M* is the moisture content at any time, and *M*_e_ is the equilibrium moisture content (g water/g dry matter). Three commonly applied empirical models shown in Table 1 were fitted to the experimental data set (MR, *t*) to describe the drying kinetics of tiger nuts. We used a nonlinear regression procedure in SPSS version 20.0 to determine the drying rate potential (*k*) and drying coefficients (*a*, *b*, *c*, and *n*) in the empirical models and performed the algorithm for estimating the model fitting using the modified Levenberg-Marquardt algorithm [12]. Given an initial value for the constant in the model, the objective function is solved. For *k* + 1, *k* = 0, 1, 2, ⋯. We computed the functions *f*(*k*)*i* = *fi*(∅(*F*)),  *R*(*k*)*i* = *yi* − *f*(*k*)*i*, *Fk* = *F*(∅(*k*)), and *J*(*k*) = *J*(∅(*k*)) and chose a positive scalar such that *F*(∅(*k*) + *hk*) < *Fk* where *hk* = −(*J*(*k*)′*J*(*k*) + *αkI*) − 1*J*(*k*)^"^*R*(*k*). The function ∅(*k* + 1) = ∅(*k*) + *hk* and *J*(*k* + 1), *R*(*k* + 1), *W*(*k* + 1), and *Fk* + 1 were computed. If any of 1 − (*Fk* − (1/*Fk*)) < *ε*1(SSCON); |*hki*/∅(*k*)*i*| < *ε*2(PCON); *k* + 1 ≥ ITER_max_; |*r*(*k* + 1)*j*| < *ε*2(RCON), where *r*(*k* + 1)*j* which is the correlation between the *j*th column *J*(*k* + 1) and *W*(*k* + 1)*R*(*k* + 1) was satisfied, then the algorithm stops and ∅∗ = ∅(*k* + 1) is predicted and the stop reason is stated else; the iteration continues.

For the judgement of the goodness of fit of the models, the following were used: the lowest reduced chi-square (*X*^2^), the root mean square error (RMSE), and the highest determination coefficient (*R*^2^) displayed in [13]

(8) $$\begin{array}{c} \chi 2=\frac{\sum_{i=1}^{N}\left({\text{MR}}_{\text{expt},i}-{\text{MR}}_{\text{pred},i}\right)2}{N-z}, \end{array}$$

(9) $$\begin{array}{c} \text{RMSE}=\sqrt{\frac{1}{N}\overset{N}{\underset{i=1}{\sum }}\left({\text{MR}}_{\text{expt},i}-{\text{MR}}_{\text{pred},i}\right)2}, \end{array}$$

(10) $$\begin{array}{c} R^{2}=\frac{N\sum_{i=1}^{N}{\text{MR}}_{\text{pred},i}{\text{MR}}_{\text{expt},i}-\sum_{i=1}^{N}{\text{MR}}_{\text{pred},i}\sum_{i=1}^{N}{\text{MR}}_{\text{expt }x,i}\;}{\sqrt{\left[N\sum_{i=1}^{N}{{\text{MR}}^{2}}_{\text{pred},i}-\left(\sum_{i=1}^{N}{\text{MR}}_{\text{pred},i}\right)2\right]\;\left[N\sum_{i=1}^{N}\text{M}{{\text{R}}^{2}}_{\text{expt},i}-\left(\sum_{i=1}^{N}{\text{MR}}_{\text{expt},i}\right)2\right]}}, \end{array}$$

where MR_expt,*i*_ is the experimental moisture ratio, MR_pred,*i*_ is the predicted moisture ratio, *N* is the number of observations, and *z* is the number of constants in the drying model.

### 2.7. Determination of Ascorbic Acid

The ascorbic acid content (AAC) of the fresh and dried tiger nut samples was determined following the protocols already reported by Kapur et al. [14] with slight modification. One gram of the sample was homogenized with a 5 ml metaphosphoric-acetic acid solution to a total volume of 10 ml. The mixture was filtered and centrifuged (Thermo Fisher Scientific, 230 V, 50/60 Hz, Waltham, Massachusetts) at 3000 rpm for 20 min, after which the supernatant was used for spectrophotometric determination. 1.54 ml of the supernatant, 90 *μ*l of bromine water, 50 *μ*l of 10% thiourea, and 390 *μ*l 2,4-dinitrophenylhydrazine solution were added in this order and incubated at 37°C for 3 hours. An ice bath was used to cool the samples for 30 min after incubation, after which 1.92 ml of chilled 85% H_2_SO_4_ was added with constant stirring. We prepared a blank and measured the absorbance at 521 nm using a spectrophotometer (T70 UV-VIS spectrophotometer, PG Instruments Ltd., Lutterworth, UK). A graph of ascorbic acid concentration versus absorbance at 521 nm was plotted (*R*^2^ = 0.9903) and used to calculate the ascorbic acid content.

### 2.8. Nonenzymatic Browning Determination

Two (2) grams of the dried samples, added to 50 ml of ethanol (60%, volume per volume), was allowed to stand for 12 hours. The mixture was stirred slowly and filtered through a 0.45 *μ*m nylon filter membrane. The supernatant browning index was estimated at 440 nm by using a spectrophotometer (T70 UV-VIS spectrophotometer, PG Instruments Ltd., Lutterworth, UK) against 60% ethanol as the blank, as reported in previous studies [15] indicating the extent of browning. The extraction was triplicated for all the samples, and the average was computed.

### 2.9. Colour Analysis

We measured the dried sample's colour in Hunter parameters (*L*∗, *a*∗, and *b*∗) with a portable colourimeter (CHN Spec, CS-10, Baoshishan, China) after calibration with standard black and white colour plates provided along with the instrument. The colour brightness coordinate *L*∗ is used to assess the whiteness, ranging from 0 (black) to 100 (white). The colourful coordinate value, *a*∗, determines green when negative and red when positive, and the chromaticity coordinate amount, *b*∗, measures yellow when positive and blue when negative [16, 17].

### 2.10. Reducing Sugars

We determined the total reducing sugars using the method reported by Negrulescu et al. [18] with a dinitrosalicylic acid reagent. One (1) gram of crushed tiger nut sample was weighed into a 50 ml flat bottom flask and extracted twice with 10 ml of hot 80% ethanol (weight per volume). We pipetted 1 ml of the solution into the test tubes, and the volume was equalized with distilled water. Three (3) millilitres of the dinitrosalicylic acid (DNS) was added to each test tube and placed in a boiling water bath for 5 min. One (1) millilitre of 40% Rochelle salt solution was added while still warm to form a dark-red colour mixture. After cooling to room temperature conditions, we measured the absorbance at 510 nm with a T70 UV-VIS spectrophotometer (PG Instruments Ltd., Lutterworth, UK). Similarly, we prepared a series of standards from the stock glucose (0.059 g in 50 ml) into concentrations of 1.180, 0.944, 0.708, 0.472, and 0.236 mg/ml. We pipetted 1 ml into a series of test tubes and added 3 ml of dinitrosalicylic acid reagent, followed by 1 ml of 40% (weight per volume) solution of Rochelle salt. We used a standard calibration curve with linearity of 0.9813 to calculate the amount of reducing sugars (RS) present in the sample using

(11) $$\begin{array}{c} \text{RS}\left(\text{mg}/100\text{ g}\right)=\frac{Cx\;V}{\text{WT}}\times D\times 100, \end{array}$$

where *C* is the concentration obtained from the calibration curve, *V* is the volume of the test tube sample, WT is the weight of the sample used, and *D* is the dilution factor.

### 2.11. Optimization of the Drying Conditions

We performed multiple response prediction for optimizing the drying condition with the response optimizer technique in Minitab, as reported in previous studies [15]. The drying conditions' goal is within the design values. We aimed at minimum amounts of drying time, moisture content, nonenzymatic browning index, yellowness, redness, and maximum values of vitamin C, brightness, and reducing sugars for the responses.

## 3. Results and Discussion

### 3.1. Effect of Tiger Nut State and Temperature on Drying Characteristic Curve

**Figure 1:**
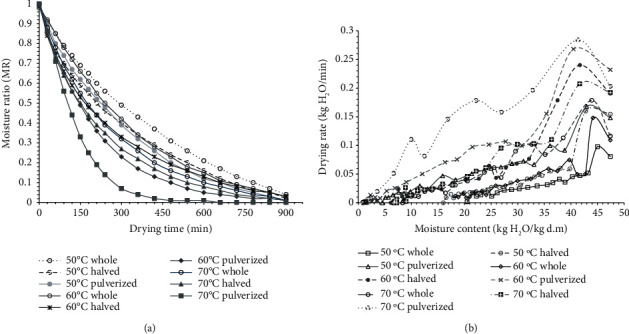
Effect of tiger nut state and air temperature on the drying characteristic curves and drying rates for the different drying conditions.

We found the initial average moisture content of the tiger nut used to be 47.43 ± 0.98% kg H_2_O/kg dry matter (32.17 ± 0.45% wet basis, w.b), which decreased to between 20.39 and 0.84 ± 0.01% kg H_2_O/kg dry matter (d.m) on dry basis (d.b) after drying at the various drying conditions for the different tiger nut states. The drying process generally followed two drying rate regimes: a constant rate in the first 3 hours and a falling rate regime after that (Figure 1(a)). As the state of the whole tiger nut is halved and pulverized, the drying rate increased significantly (*p* < 0.05), leading to an accelerated drying process and increased energy efficiency. At a drying temperature of 50°C, we observed the equilibrium moisture content (EMC) to be 20.39, 16.05, and 4.53 kg H_2_O/kg d.m for the whole, halved, and pulverized tiger nuts, respectively. As the temperature increased to 60°C, the EMC was 13.25 for the whole, 10.26 for the halved, and 1.49 kg H_2_O/kg d.m for the pulverized tiger nuts. The EMC further decreased to 8.38%, 6.71%, and 0.84% kg H_2_O/kg d.m when the air temperature was at the highest, i.e., 70°C. As the state of the tiger nut was made smaller, more moisture was removed and, in the end, resulted in a reduction in drying time and moisture content (Figure 1). To reach a moisture content of 20.39% (d.b), the drying time for the samples dried at 50°C air temperature reduced from 960 to 360 min for the intact and the pulverized tiger nut, respectively. As the air temperature increased from 50 to 70°C, we observed a similar decrease in drying time and sample moisture content. Overall, the pulverized samples dried at 70°C recorded the lowest moisture content and the highest drying rate (Figure 1(b)).

In comparison, the intact tiger nut dried at 50°C retained the highest moisture content and the lowest drying rate. This occurrence suggests significant savings in time as the state of the intact tiger nut is manipulated, and the air temperature increased [19]. We observed that the drying rate at the initial stages of drying increased sharply, and after that, it slowed (Figure 1(b)). Compared with the intact tiger nut, splitting and pulverization enhanced the heat and mass transfer within the sample tissues resulting in improved drying rates and energy utilization [9]. The increase in the surface area due to splitting and pulverization may have caused the accelerated drying rate to result in low moisture content associated with these samples. The decrease in drying time and moisture content with an increase in the surface area and air temperature has been reported for other food materials, including orange fleshed sweet potato slices [20] and cassava chips [21].

### 3.2. Effect of Tiger Nut State and Temperature on Moisture Diffusivity and Activation Energy

**Figure 2:**
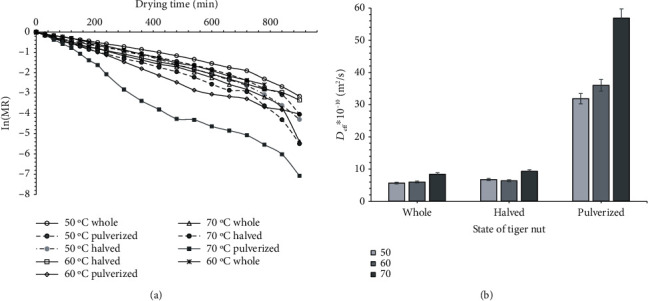
Variation of In(MR) against drying time and the effective moisture diffusivity (*D*_eff_) for tiger nuts dried at the various conditions.

We used the variation of In(MR) against the drying time graph for the various drying conditions to calculate the effective moisture diffusivity *D*_eff_ displayed in Figure 2(a). The effect of the state of the tiger nut and air temperature on *D*_eff_ is apparent (Figure 2(a)). In general, we found a linear regression with a negative slope for In(MR) against the drying time at the initial stages of drying. However, we did not see this linearity in the later stages of drying. The values of the correlation coefficient ranged from 0.9253 to 0.978. The high correlation coefficient indicates adequate predicting capacity between the actual and the simulated values. The effective moisture diffusivity increased with manipulation of the tiger nut state and increasing the drying air temperature. However, there were no significant differences in the intact and halved samples. At a drying temperature of 50°C, the effective moisture diffusivity increased from 5.67 × 10^−10^ m^2^/s for the whole tiger nut to 31.88 × 10^−10^ m^2^/s pulverized samples. We observed a similar increasing trend from 6.0 × 10^−10^ m^2^/s to 36.0 × 10^−10^ m^2^/s and from 8.42 × 10^−10^ m^2^/s to 56.9 × 10^−10^ m^2^/s for the whole and the pulverized samples dried at 60 and 70°C, respectively. The results agree with *D*_eff_ values reported for quince [13] and almond kernels [22]. Generally, *D*_eff_ increased as the intact tiger nut was halved or crushed. *D*_eff_ for the crushed pieces was higher than that for the halved ones. The moisture removal rate between the intact and the halved tiger nuts was far lower than that in the pulverized ones (Figure 2(b)). The crushed samples' effective moisture removal rate increased between 5.6- and 6.75-fold at the different air temperatures compared to that of the intact tiger nuts.

We observed that at the initial stages of drying, the product's temperature rose sharply due to more heat absorption. At this stage, the sample's moisture diffuses rapidly through the pieces' pores; thus, there is a high loss factor at higher moisture content. This movement increases the water vapour pressure inside the pores and results in pressure-inducing pore openings in the tiger nut's tissues [23]. The *D*_eff_ amounts recorded in this study for tiger nut is within the broad range of 10^−8^–10^−12^ m^2^s^−1^ to dry food materials [24]. *D*_eff_ recorded in this study is relatively higher than hot-air drying of dika kernels and nuts [23] but lower than ultrasonic hot-air drying of walnuts [25].

**Figure 3:**
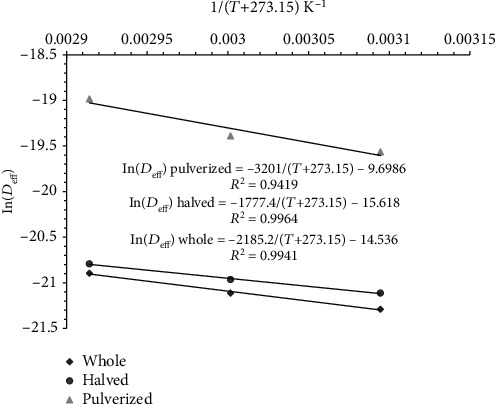
Variation of In(*D*_eff_) against 1/(*T* + 273.15) for the whole, halved, and pulverized tiger nuts.

The *D*_eff_ values were used to fit equation (6) to estimate the activation energy, *E*_a_, for moisture diffusion.  Figure 3 illustrates the variation of In(*D*_eff_) against 1/(*T* + 273.15) for the whole, halved, and pulverized tiger nut. Such a fitting gave regression coefficients 0.94 and 0.99, showing that the goodness of such a fitting was satisfactory. *E*_a_ was 18.17 kJ/mol for the intact, 14.78 kJ/mol for the halved, and 26.61 kJ/mol for the pulverized tiger nut samples. These *E*_a_ values suggest that drying the crushed tiger nuts to equilibrium moisture content requires higher energy than drying the intact and the halved nuts. This is expected because adequate moisture removal was achieved in the pulverized samples. *E*_a_ obtained for the unbroken and halved tiger nuts was closer to that of dika kernels (16.75 kJ/mol) [17, 24], walnut kernels (18.04-28.8 kJ/mol) [25], while that of the mashed tiger nut was similar to the activation energy of cashew kernels (28.7 kJ/mol) [26]. The activation energy amounts obtained in this study were lower than 37.02 kJ/mol [17, 24] and 51.26 kJ/mol [27] for drying dika nuts and okra, respectively. The activation energy for moisture removal during drying depends highly on the chemical structure and the moisture content of the initial sample [25, 28].

### 3.3. Modelling of the Drying Curves

We fitted the dimensionless moisture ratio, MR, and drying time, *t*, data set to the page, logarithmic, and Midilli et al. empirical models using nonlinear regression analysis to select a suitable mathematical model for convective hot-air drying of tiger nuts.  Table 2 shows the results of such a fitting to the experimental data for the various modified tiger nut samples and their estimated constants with their corresponding statistical parameters characterizing each fitting for the different drying temperatures. From the results obtained, it is evident that the experimental data fitted well with the models tested in this study. The relatively high correlation coefficients (*R*^2^), low reduced chi-square (*χ*^2^), and root mean square errors (RMSE) indicate a satisfactory predicting capacity for the conditions used over the entire drying process.

The results obtained from the convective hot-air drying experiments indicated that the *R*^2^ values for the three models were all above 0.992. The statistical parameter estimations showed that the root mean square error (RMSE) and the reduced chi-square (*χ*^2^) amounts ranged from 0 to 0.02627 and 0 to 0.0005, respectively, evincing that all the three models tested could be adequately used to predict convective hot-air drying of tiger nuts. The relatively high correlation coefficients, low reduced chi-square, and root mean square errors indicate an adequate predicting capacity for the drying conditions studied [25]. We found the Midilli et al. model to be the most suitable thin-layer drying model among the models examined for convective hot-air drying of tiger nuts with the highest value for the coefficient of determination (*R*^2^) and lowest reduced chi-square (*χ*^2^) and RMSE.

**Figure 4:**
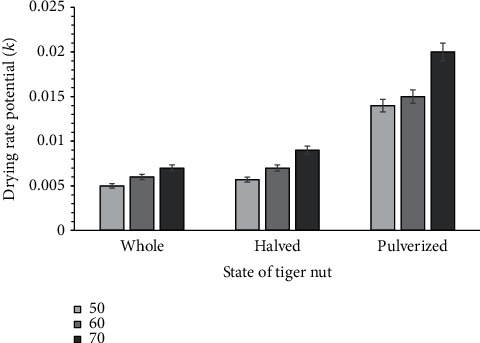
Drying rate constant (*k*) for the Midilli et al. model for the tiger nuts dried at the various conditions.

The Midilli et al. drying rate constant *k* for the drying conditions studied showed that *k* predominantly increased with increased air temperature and the modification of the unbroken tiger nuts to increase its surface area. This behavior implies that the tiger nut samples' drying rate potential generally increased with increased air temperature and splitting into two or crushing into coarse particles. At a drying temperature of 70°C, breaking the intact tiger nuts enhanced the drying rate potential from 0.007 to 0.009 kg H_2_0 per second (Figure 4). The drying potential was 0.020 kg H_2_0 per second when undamaged tiger nut was crushed. An increase in temperature from 50 to 70 indicated an increase in drying rate potential, but the other constants in the drying models did not show any definite trend. This indefinite trend is similar to mathematical drying modelling for dika nuts and kernels [24].

### 3.4. Effect of Drying Conditions on Tiger Nut Quality

#### 3.4.1. Reducing Sugars and Browning

Table 3 displays the drying conditions and the results of the drying time, moisture content, reducing sugars, vitamin C, browning index, colour parameters, and the effective moisture diffusivity for dried tiger nuts.

**Figure 5:**
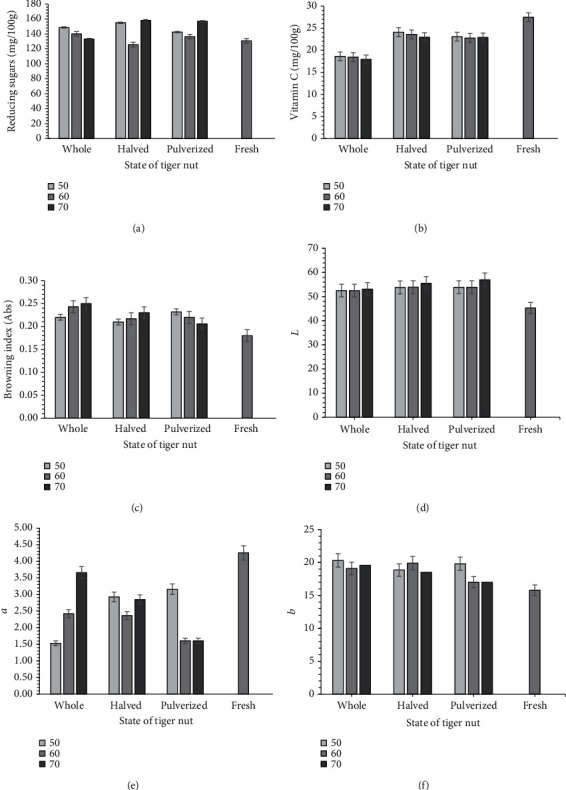
Effect of drying conditions on tiger nut quality.

Reducing sugar is another quality criterion for dried tiger nut snack and milk beverage preparation. Sugars that are capable of acting as reducing agents because of their free aldehyde or ketone group is called reducing sugars. In bakery products, the reducing agents modify the rheological properties of doughs and batters to cause the aggregation of hydrogen atoms to reactive sites of the molecules. In the reaction process, it weakens the gluten structure in the dough by breaking the intra- and intermolecular covalent bonds between the proteins [3]. This mechanism is essential for mixing high-protein flours in a short time to reduce the energy requirements for the kneading process. High reducing sugars are desirable for other food applications, including yoghurt, jam, beer, liqueur, and pastry production. The impact of the state of tiger nut and air temperature on the reducing sugars of tiger nut was significant (*p* < 0.05) as shown in Figure 5(a) and Table 4, showing the relative contribution of the factors to the responses and their *p* values. After drying at the various drying conditions, the tiger nut's reducing sugars increased from 130.8 mg/100 dry weight in the raw tiger nuts to between 133.11 and 158.18 mg/100 dry weight. For the whole tiger nuts, an increase in air temperature reduced the amounts of reducing sugars, but in the halved and pulverized ones, we observed no definite trend in the reducing sugars. The crushed and split samples dried at 70°C recorded the highest reducing sugars while the ones dried at 60°C recorded the lowest. Tiger nuts were dried during storage, and in storage, reducing sugars have been reported to increase [2]. In this study, the browning index for the dried samples was more elevated than that for the fresh ones. The browning index increased from 0.18 absorbance (Abs) unit to between 0.21 and 0.25 Abs unit after dehydration at the various conditions of tiger nuts. The browning index in the uncut and split samples generally increased with temperature, while the pulverized ones were reduced but not significant. Browning in food during drying is a complex phenomenon, but browning's overall effect reduces dried foods' nutritional value. The carbonyl groups' reactions of reducing sugars and amino groups of amino acids form brown pigments, usually called the Maillard reaction. Oxidation of vitamin C has also been found to results in nonenzymatic browning of food. This oxidation commences with the formation of dehydroascorbic acid, which further reacts with amino compounds to form brown pigments [29]. Cernisev found nonenzymatic browning caused by temperature to follow a zero-order response for temperature below 70°C [30]. Beyond the browning index of 0.6 Abs unit, consumers did not find dried products desirable, depicting that all the dried samples are acceptable.

#### 3.4.2. Ascorbic Acid

The experiment results show that tiger nuts' form and air temperature significantly (*p* < 0.05) affected the dried tiger nuts' vitamin C content. The vitamin C in the intact dried samples ranged from 17.93 to 18.62 mg/100 dry weight, whereas the halved and the pulverized samples' vitamin C content ranged from 22.96 to 24.09 mg/100 g and 22.90 to 23.08 mg/100 g dry weight, respectively (Figure 5(b)). Compared to the 27.46 mg/100 dry weight vitamin C content in the fresh sample, all the dried tiger nuts degraded between 12 and 35% of the vitamin C in the fresh tiger nuts. The degradation rate was highest in the uncut tiger nut (32-35%), while there was a similar degradation rate for the halved and the pulverized samples (12-17%). As the air temperature increased from 50 to 70°C, the vitamin C generally decreased from 18.62 to 17.93 mg/100 g dry weight, 24.09 to 22.96 mg/100 g dry weight, and 23.08 to 22.90 mg/100 dry weight for the respective whole, halved, and pulverized tiger nuts. The values recorded in this study are similar to vitamin C content reported for dried chestnuts (21.8 mg/100 g), dried Japanese chestnut (17.4 mg/100 g), and dried Chinese chestnuts (16.6 mg/100 g) [31]. In yam drying at 90°C, Abano and Amoah observed 88% reduction in vitamin C content [32]. Barros and others [33] reported between 25 and 54% of vitamin C losses during boiling and 2 to 77% during roasting of chestnuts. Vitamin C is heat-sensitive and very reactive and degrades mainly by thermal or oxidative means. Oxidative vitamin C degradation primarily occurs due to long drying times arising from increased sample thickness and tough skin, preventing moisture removal from the food product's surface [20]. Oxidative vitamin C degradation due to the intact samples' long drying times may have caused such declines in ascorbic acid.

#### 3.4.3. Colour Parameters

The colour of dried food is an essential consideration in food products because colour and general appearance are generally the first impressions consumers have about a specific product [7]. The closer it is to the fresh, the more it is desired. The brightness, redness, and yellowness colour results for the fresh, whole, halved, and pulverized tiger nuts are displayed in Figures 5(d)–5(f), respectively. Generally, the dried tiger nuts at the different forms and temperatures were brighter than the fresh ones. However, the halved and the pulverized samples' appearance was more colourful than that of the unbroken ones. The brightness of the whole tiger nuts increased from 52.1 to 53.12, while the split and the mashed ones increased from 53.90 to 55.53 and 53.90 to 56.97. Similarly, the yellowness increased after drying from the fresh (15.80) to between 17.0 and 20.33 for the different forms and air temperatures studied. Mostly, as the air temperature increased from 50 to 70°C, the yellowness decreased. The redness of the dried tiger nuts, on the other hand, reduced after dehydration. Hot air temperature and the state of the tiger nut were not significant (*p* > 0.05) on the brightness and yellowness of the dried nuts.

The fresh redness (4.26) reduced to between 1.53 and 3.66 when dried at the various air temperatures. The air temperature increase did not follow any definite trend in the samples' redness. Reduction in redness is usually attributed to the amino acids' reaction and reducing sugars (Maillard) in the tiger nut tubers during dehydration. Coşkuner et al. [2] reported the colour parameters of tiger nuts. They analyzed the brightness (*L*∗), redness (*a*∗), and yellowness (*b*∗) of the whole and pulverized tiger nut and reported *L*∗ = 31.99, *a*∗ = 7.01, and *b*∗ = 10.89 in the whole and *L*∗ = 58.78, *a*∗ = 16.20, and *b*∗ = 16.20 in the crushed tubers. Consistent with our study results, Coşkuner and others [2] found the intact tiger nut to be darker than the ground tiger nut and *a*∗ and *b*∗ values to be lower. Optimization of the drying parameters is performed.

We predicted the optimal conditions for convective hot-air drying of tiger nuts with the composite desirability concept in Minitab statistical software. The results simulated with 95% confidence in the range of factors used in the study gave the optimal form of tiger nuts as pulverized or crushed and drying air temperature as 70°C. Nonsignificant responses were dropped in the optimization analysis. At this drying condition, the predicted drying time, moisture content, vitamin C, reducing sugars, browning index, brightness, redness, and yellowness are 780 min, 0.84 kg H_2_0/kg dry matter, 22.9 mg/100 mg dry weight, 157.01 mg/100 g dry weight, 0.21 Abs unit, 56.97, 1.6, and 17.0, respectively. We crushed fresh tiger nut samples and verified the predicted optimal conditions. The simulated results were closer to the verified actuals: 780 min for drying time, 4.64 kg H_2_0/kg for moisture content, 20.19 mg/100 mg dry weight for vitamin C content, 159.81 mg/100 g for reducing sugars, 0.25 Abs unit for the browning index, 73.96 for brightness, 3.13 for redness, and 17.32 for yellowness. This high predicting capacity by the optimization studies shows the accuracy and the goodness of fit. The composite desirability for this optimal condition was 0.965.

## 4. Conclusion

The state of tiger nut before drying was significantly affected by the final moisture and vitamin C content. Manipulation of the intact tiger nut and increase in air temperature substantially reduced the drying time, enhanced the effective moisture removal, and accelerated the drying rate. The effective moisture removal rate was highest for the pulverized samples, followed by the split and the intact tiger nut tubers. The drying rate potential was significantly affected by the state of the tiger nut and the air temperature for all the drying models tested. Pulverization of the tiger nut before convective hot-air drying could potentially save more than 27.8% drying time and result in a product with low moisture content for safe storage. Among the thin-layer drying models tested, the Midilli et al. model best described the convective hot-air drying of tiger nuts. The products' quality attributes in terms of brown pigment formation, brightness, and reducing sugars improved with pulverization as compared to those of the unbroken ones. Vitamin C degradation was higher in the intact tiger nuts than in the pulverized samples. For optimum drying kinetics and quality of tiger nut for the flour industry, the authors recommend pulverization before convective hot-air drying at 70°C. Further studies on the influence of hot air and tiger nut state on antinutrients are recommended.

## Figures and Tables

**Table 1:** Mathematical models that were applied to drying data.

Model name	Model expression	References
Page	MR = exp(−*kt*^*n*^)	[9]
Logarithmic	MR = *a*exp(−*kt*) + *c*	[2]
Midilli et al.	MR = *a* exp(−*kt*^*n*^) + *bt*	[9]

**Table 2:** Results of fitting the experimental data for tiger nuts and their estimated constants for the various air temperatures.

Model name	*T* (°C)	State	*a*	*k*	*n*	*b*/*c*	*R* ^2^	RMSE	*X* ^2^

Page	70	Whole		0.005	0.972		0.998	0.012247	0.000167
		Halved		0.007	0.922		0.998	0.012247	0.000167
		Pulverized		0.012	1.234		0.999	0.01	0.000111
	60	Whole		0.002	1.113		0.998	0.014142	0.000222
		Halved		0.009	0.85		0.997	0.014142	0.000222
		Pulverized		0.011	1.011			0.01	0.000111
	50	Whole		0.002	1.077		0.993	0.02582	0.000737
		Halved		0.005	0.922		0.992	0.026726	0.000789
		Pulverized		0.010	1.05		0.996	0.019518	0.000421

Midilli et al.	70	Whole	1.014	0.007	0.903	−3.79*E* − 05	0.999	0.01	0.000125
		Halved	1.006	0.009	0.865	−3.62*E* − 05	0.999	0.007071	6.25*E* − 05
		Pulverized	0.997	0.02	1.266	5.60*E* − 06	0.999	0.01	0.000125
	60	Whole	0.984	0.006	1.094	−3.48*E* − 05	0.999	0.01	0.000125
		Halved	1.008	0.007	0.774	−5.71*E* − 05	0.998	0.01	0.000125
		Pulverized	0.986	0.015	1.037	2.83*E* − 06	0.999	0.01	0.000125
	50	Whole	0.999	0.005	0.855	0.00*E* + 00	1	0	0
		Halved	0.997	0.0057	0.736	0.00*E* + 00	1	0	0
		Pulverized	0.993	0.014	0.946	−8.13*E* − 05	0.999	0.006901	5.88E-05

Logarithmic	70	Whole	0.996	0.004		-0.004	0.998	0.014142	0.000235
		Halved	0.97	0.004		0.004	0.997	0.014142	0.000235
	60	Pulverized	1.062	0.007		-0.012	0.994	0.024495	0.000706
		Whole	1.086	0.003		-0.087	0.999	0.012247	0.000176
		Halved	0.926	0.004		0.029	0.994	0.022361	0.000588
		Pulverized	0.999	0.005		-0.005	0.999	0.01	0.000118
	50	Whole	1.154	0.002		-0.184	0.999	0.009759	0.000111
		Halved	0.992	0.003		-0.057	0.995	0.020702	0.0005
		Pulverized	1.052	0.005		-0.074	0.999	0.009759	0.000111

**Table 3:** Drying conditions and results of drying time, moisture content, reducing sugars, vitamin c browning index, colour parameters, and effective moisture diffusivity for dried tiger nuts.

State of tiger nut	*T* (°C)	DT (min)	MC (% d.b)	RS (mg/100 g)	Vit C (mg/100 g)	BI (Abs unit)	*L*∗	*a*∗	*b*∗	*D*_eff_ (m^2^/s) *E* − 10
Halved	60	960	10.26	125.79	23.58	0.19	56.97	2.37	19.92	6.40
Halved	50	1080	16.02	154.89	24.09	0.21	50.81	2.93	18.86	6.77
Whole	70	960	8.38	133.11	17.93	0.18	53.12	3.66	19.57	8.42
Pulverized	70	780	0.84	157.01	22.9	0.21	53.9	1.6	17	41.59
Whole	50	1080	20.4	148.61	18.62	0.21	52.51	1.53	20.33	5.67
Halved	70	900	6.71	158.18	22.96	0.19	52.07	2.85	18.53	9.33
Pulverized	50	1080	4.53	142.6	23.08	0.24	55.53	4.16	19.81	31.88
Pulverized	60	960	1.49	136.24	22.78	0.25	53.9	1.6	17	36.0
Whole	60	960	13.25	140.12	18.43	0.22	52.51	2.42	19.1	6.00

*T*: air temperature; DT: drying time; MC: moisture content; RS: reducing sugars; Vit C: vitamin C; BI: nonenzymatic browning index; *L*∗: whiteness; *a*∗: redness; *b*∗: yellowness; *D*_eff_: effective moisture diffusivity.

**Table 4:** Model and factor contribution to responses and their significance.

Parameter	Factor contribution (%) with *p* values in brackets			
	Model	Temperature	State of tiger nut	State∗ temperature
DT (min)	85.57 (0.006)	78.35 (0.024)	7.22 (0.444)	14.43 (0.45)
MC (% dry basis)	94.80 (0.008)	30.79 (0.021)	64.01 (0.006)	5.20 (0.06)
BI (Abs unit)	57.49 (0.024)	2.05 (0.655)	13.39 (0.086)	42.10 (0.011)
Ascorbic acid (Vit C) (mg/100 g)	97.95 (<0.0001)	7.26 (<0.0001)	65.33 (<0.0001)	25.36 (<0.0001)
Reducing sugars (mg/100 g)	99.21 (<0.0001)	34.25 (<0.0001)	41.09 (<0.0001)	23.86 (<0.0001)
*L*∗	30.63 (0.4730)	4.84 (0.545)	3.29 (0.659)	22.50 (0.256)
*a*∗	79.07 (<0.0001)	7.49 (0.064)	0.75 (0.727)	70.83 (<0.0001)
*b*∗	39.93 (0.227)	17.76 (0.097)	12.86 (0.175)	9.31 (0.604)
*D*_eff_ (m^2^/s)	93.13 (0.002)	6.57 (0.261)	86.56 (0.002)	6.87 (0.563)

## Data Availability

The data that support the findings of the study are available within the manuscript.
